# Sleep, Fatigue, and Depressive Symptoms among Female Nurses with Allergic Rhinitis

**DOI:** 10.3390/healthcare9101328

**Published:** 2021-10-05

**Authors:** Oksoo Kim, Bohye Kim, Hyunseon Jeong, Jisun Lee, Heeja Jung

**Affiliations:** 1 College of Nursing, Ewha Womans University, 52, Ewhayeodae-gil, Seodaemun-gu, Seoul 03760, Korea; ohong@ewha.ac.kr (O.K.); bohyekim516@naver.com (B.K.); idadoshi@hotmail.com (H.J.); rollorcoaste@naver.com (J.L.); 2 College of Nursing, Konyang University, 158, Gwanjeodong-ro, Seo-gu, Daejeon 35365, Korea

**Keywords:** rhinitis, sleep, fatigue, depression, nurses, woman, shift work

## Abstract

Allergic rhinitis (AR) is a common chronic disease that negatively affects physical and mental health. The purpose of this study was to determine the effects of allergic rhinitis on sleep, fatigue, and depressive symptoms among Korean female nurses. This was a cross-sectional study conducted using data from the Korea Nurses’ Health Study (KNHS), and a total of 8645 female nurses was selected for the final analysis. The demographic characteristics, Body Mass Index, alcohol consumption, shift work, comorbidities (atopic dermatitis and asthma), self-rated health, sleep disturbance (Jenkins Sleep Questionnaire), fatigue (Chalder Fatigue Scale), and depressive symptoms (Perceived Health Questionnaire-9) were collected. The data were analyzed using chi-square tests, *t*-tests, and hierarchical multiple regression analyses. Participants with allergic rhinitis had significantly greater sleep disturbance, fatigue, and depressive symptoms than those without allergic rhinitis, and allergic rhinitis was a significant factor influencing sleep disturbance and fatigue among the participants after controlling for confounding variables. Therefore, it is imperative to develop effective interventions to manage allergic rhinitis symptoms and improve sleep and fatigue in affected nurses.

## 1. Introduction

Allergic rhinitis (AR) is a common chronic disease affecting 10–40% of the population worldwide [1]. In the United States, 7.7% of adults are diagnosed with AR each year [2], while the prevalence of AR among Korean adults is 14.6% [3]. Exposure to hazardous substances in the workplace may increase the risk of developing AR for healthcare workers [4]. The incidence of AR among nurses has been reported to be 20% in Pakistan [5]; in Japan, 10.7% of nurses have asthma; among whom, the incidence of AR was reported to be 54.3% [6]. AR is a chronic disease that negatively affects sleep, quality of life, and work performance [7,8], resulting in high healthcare and social costs [9].

The main symptoms of AR are nasal itching, sneezing, rhinorrhea, and nasal congestion [10], with nasal obstruction being the symptom most often experienced by AR patients [11]. According to previous studies, adults with AR experience obstructive sleep apnea and sleep-disordered breathing [12,13], as well as prolonged sleep latency, insomnia, sleep disturbances, and daytime dysfunction [14]. In addition, the severity of nasal congestion was reported to be highest during the night and early morning, with increased daytime sleepiness and fatigue [15]. Some studies have reported that patients with seasonal allergies experienced more fatigue than healthy adults [16]. Park et al. [17] suggested that patients with AR and obstructive sleep apnea have a higher level of fatigue than those with only obstructive sleep apnea.

AR can affect mental health, as well as physical health. In adults with AR, the more persistent and severe the symptoms, the higher the degree of depression [17,18,19]. Grosso et al. [20] reported that depression was associated with the failure to control AR symptoms and limitations in daily activities due to the disease. Meanwhile, in another study, a depressed mood was associated with decreased productivity rather than with symptoms of AR [21]. Therefore, the relationship between AR and depression remains unclear, and further research is needed.

Few studies have investigated the effects of AR on physical and mental health specifically among nurses, who are frequently exposed to workplace allergens. Sleep, fatigue, and depression, which have been reported to be related to AR, can negatively affect nurses’ job performances. Therefore, this study was conducted to determine the effect of AR on sleep disturbance, fatigue, and depressive symptoms among a nationwide cohort of Korean nurses.

## 2. Materials and Methods

### 2.1. Study Design, Population, and Setting

This study is a cross-sectional study conducted using data from the Korea Nurses’ Health Study (KNHS), an ongoing web-based cohort study started to identify factors affecting women’s health. KNHS, which started in 2013, uses a similar protocol as the NHS3 in the United States but uses a questionnaire customized for Korea [22]. Participants in the Module 1 survey, which was the KNHS baseline survey, included 20,613 female nurses in their reproductive years (i.e., aged 20–45 years), and these participants were working within one year of the baseline survey. A total of 10,253 female nurses participated in the KNHS Module 8 survey from October 2019 to April 2020. This study targeted 8645 female nurses who participated in the Module 8 survey, except not employed women for 1 year.

### 2.2. Measures

Sleep disturbance was measured using Jenkins Sleep Questionnaire (JSQ) [23]. The tool consists of four items asking participants how many days they have experienced sleep problems in the previous 30 days. The questions include difficulty falling asleep, waking up several times at night, difficulty falling asleep again after waking up at night, and feeling tired after the usual amount of sleep. Each question is measured on a 6-point Likert scale from 0 to 5: “Not at all (0)”, “1−3 days (1)”, “4−7 days (2)”, “8−14 days (3)”, “15−21 days (4)”, or “22−30 days (5)”. The possible range of scores is 0−20, and the higher the total score, the higher the degree of sleep disturbance. Cronbach’s alpha of the original study was reported to be 0.79 [23], and the Cronbach’s alpha of this study was 0.85.

Fatigue was measured using the Chalder Fatigue Scale (CFS) [24]. This tool consists of 11 items and includes questions about physical changes due to fatigue, decreased thinking ability, and decreased memory. The CFS consists of two subcategories, 7 items for physical fatigue and 4 items for mental fatigue. Each item is measured on a 4-point Likert scale from 0 to 3: “better than usual (0),” “no more than usual (1),” “worse than usual (2)”, or “much worse than usual (3)”. The possible range of total scores is 0–33, with 0–21 for physical fatigue and 0–12 for mental fatigue. The higher the score, the higher the level of fatigue. The Cronbach’s alpha of the original tool was 0.90 [24] and that of this study was 0.90.

Depressive symptoms were measured using the Patient Health Questionnaire (PHQ-9) [25]. The 9-item PHQ-9 measures displeasure, fatigue, appetite change, guilt or worthlessness, decreased concentration, slow movement or restlessness, and suicidal ideation. Each item is measured on a 4-point Likert scale: “not at all (0)”, “several days (1)”, “more than half of the days (2)”, or “nearly every day (3)”. The possible range for the total score is 0–27, and the higher the score, the higher the degree of depressive symptoms. The Cronbach’s alpha of the original tool was reported as 0.86 [25], and the Cronbach’s alpha in this study was 0.86.

Baseline characteristics, comorbidities, and self-rated health were included as covariates based on a literature review to determine the effect of AR on sleep disturbance, fatigue, and depressive symptoms. The baseline characteristics included age, education level, marital status, body mass index (BMI), alcohol consumption, and shift work. The comorbidities included atopic dermatitis and asthma. Sleep disturbance, fatigue, and depressive symptoms have been reported to affect each other [26,27,28], so they were included as confounders in each regression model.

### 2.3. Ethical Considerations

This study was conducted after approval by the institutional review board for the ethical protection of participants (IRB No. 201904-0012-03). Anonymity and confidentiality were assured, and informed consent was obtained from the participants as part of the online survey.

### 2.4. Data Analysis

A data analysis was performed using SPSS version 26.0 (IBM Corp., Armonk, NY, USA). The chi-square test and *t*-test were used to analyze and confirm the differences in the baseline characteristics and comorbidities between the AR group and the non-AR group. The *t*-tests were used to check the differences in the degree of sleep disturbance, fatigue, and depressive symptoms between the AR group and the no AR group.

Finally, hierarchical multiple regression analyses were performed to examine the effect of AR on the sleep disturbance, fatigue, and depressive symptoms. Hierarchical multiple regression was utilized using four stage models. The stage 1 model included the AR status. The stage 2 model included age, level of education, marital status, BMI, alcohol consumption, and shift work, while the stage 3 model included atopic dermatitis and asthma as the confounding variables. In stage 4, each model included sleep disturbance, fatigue, or depressive symptoms as the confounders, as well as self-rated health. The threshold of statistical significance for this study was *p* < 0.05.

## 3. Results

### 3.1. Baseline Characteristics according to Allergic Rhinitis Status

Of the 8645 study participants, the prevalence of AR was 20.9% (*n* = 1808). There were significant differences in the age (χ^2^ = 10.16, *p* = 0.017), atopic dermatitis (χ^2^ = 206.69, *p* < 0.001), asthma (χ^2^ = 252.02, *p* < 0.001), and self-rated health (χ^2^ = 44.70, *p* < 0.001) between the AR and the no AR groups (Table 1). In the AR group, the largest proportion of participants (59.1%) was in the 30–39 age group. In the AR group, 15.2% of the participants had atopic dermatitis and 7.0% had asthma, which were both higher compared to the no AR group. The participants who rated their health as poor were 17.0% in the AR group and 12.4% in the no AR group, indicating that the level of self-rated health was lower in the AR group (Table 1).

### 3.2. Sleep Disturbance, Fatigue, and Depressive Symptoms among Participants with and without Allergic Rhinitis

Table 2 shows the differences in sleep disturbance, fatigue, and depressive symptoms between the AR group and the no AR group. Significant differences were found in the sleep disturbance, fatigue, and depressive symptoms according to the AR status. The mean score of sleep disturbance in the AR group was 5.42 (SD = 4.81), which was higher than that of the no AR group (t = −7.28, *p* < 0.001). The mean score of total fatigue in the AR group, 17.44 (SD = 5.74), was also higher than that of the no AR group (t = −8.39, *p* < 0.001). Participants with AR had significantly higher depressive symptoms (M ± SD = 5.41 ± 4.33) than those without AR (t = −5.48, *p* < 0.001) (Table 2).

### 3.3. The Effect of Allergic Rhinitis on Sleep Disturbance, Fatigue, and Depressive Symptoms

Hierarchical multiple regression analyses were performed to identify the effect of allergic rhinitis on the sleep disturbance, fatigue, and depressive symptoms (Table 3, Table 4 and Table 5). The VIFs were 10 or less, and the tolerance value range 1.0 or less in all of the models; therefore, there was no problem with multicollinearity.

From the multiple regression analyses, AR was identified as a significant factor influencing sleep disturbance (β = 0.03, *p* < 0.001) and fatigue (β = 0.03, *p* < 0.001) among the participants after controlling for confounders. However, AR was not found to have an influence on depressive symptoms. The explanatory power of model 4 was 35.4% (F = 339.81, *p* < 0.001) for sleep disturbance and 46.3% (F = 533.39, *p* < 0.001) for fatigue.

Among the control variables, the factors affecting sleep disturbance were age (β = −0.03, *p* = 0.001), alcohol consumption (β = 0.02, *p* = 0.016), shift work (β = 0.07, *p* < 0.001), atopic dermatitis (β = 0.03, *p* < 0.001), self-rated health (fair β = 0.04, *p* < 0.001; poor β = 0.07, *p* < 0.001), fatigue (β = 0.09, *p* < 0.001), and depressive symptoms (β = 0.48, *p* < 0.001). The confounders affecting fatigue were marital status (β = −0.11, *p* < 0.001), BMI (β = −0.07, *p* < 0.001), shift work (β = 0.02, *p* = 0.003), self-rated health (fair β = 0.20, *p* < 0.001; poor β = 0.22, *p* < 0.001), sleep disturbance (β = 0.08, *p* < 0.001), and depressive symptoms (β = 0.52, *p* < 0.001). Lastly, age (β = −0.05, *p* < 0.001), marital status (β = 0.11, *p* < 0.001), BMI (β = 0.04, *p* < 0.001), self-rated health (poor β = 0.06, *p* < 0.001), sleep disturbance (β = 0.35, *p* < 0.001), and fatigue (β = 0.46, *p* < 0.001) increased the risk of depressive symptoms (Table 3, Table 4 and Table 5).

## 4. Discussion

The present study investigated the prevalence of AR among Korean female nurses to determine the associations between AR and sleep disturbances, fatigue, and depressive symptoms by analyzing the data from the Korea Nurses’ Health Study. The results revealed that the prevalence of AR among Korean female nurses was 20.9%, which was slightly lower than the 24.5% prevalence among Korean females aged 20–29 years old that was identified through data from the 5th Korea National Health and Nutrition Examination Survey (2010−2012) [29]. However, considering that the prevalence of AR has been found to peak between 20 and 29 years of age and then gradually decrease [29], it may be that the prevalence of AR was relatively low in this study because females aged 30 years or older accounted for 82.1% of the participants.

Few studies have been conducted on the prevalence of AR among nurses. A study involving nurses at university hospitals in Thailand reported the prevalence of AR among nurses to be 7.9% [30], and a study involving healthcare workers at university hospitals reported the prevalence of AR among healthcare workers to be approximately 20% [5]. Both rates were lower than that of the present study. However, because the prevalence of AR can vary greatly depending on sex, race, and age [29], further research is needed to determine and compare the prevalence of AR in female nurses of varying races and ages.

In the present study, when the confounding variables were controlled, AR was significantly associated with sleep disturbances. Previous studies on the associations between AR and sleep disturbances have shown relatively consistent results. A study in the United States involving 5563 adults aged 18 years or older demonstrated a significant association between AR and poor sleep parameters, including insomnia [14]. In addition, a large Spanish cohort study involving adults aged 18 years or older found that AR symptoms were significantly associated with poor sleep quality [19]. Nurses have poor sleep quality due to the nature of their work compared to those in other occupations [31], and AR symptoms can worsen nurses’ sleep problems. Sleep disturbances can weaken the concentration during the performance of nursing tasks, lower the productivity of nursing care, and negatively affect the quality of nursing care. Therefore, individual efforts, in addition to organizational attention and support for the management of AR symptoms among nurses, are needed.

The results of the present study revealed that, with the confounding variables controlled, AR was significantly associated with fatigue. This result is similar to the results of previous studies regarding the association between AR and fatigue. Specifically, one study on the association between AR and fatigue among patients with obstructive sleep apnea (OSA) [17] showed that fatigue was significantly higher in the OSA–AR group compared with the OSA group. In addition, another study on the effects of AR treatment on sexual ability and fatigue [32] found that the AR treatment reduced fatigue from 62.5% to 22.9% in female subjects and from 54.2% to 35.4% in male subjects. These results suggest that having AR may be a major factor in the experience of fatigue and that the ineffective management of AR symptoms may worsen fatigue among nurses who already have higher mental and physical fatigue due to the nature of their work [33]. The studies conducted on the association between AR and fatigue among nurses have been homogeneous. However, considering that the high prevalence of AR and high levels of fatigue among nurses significantly affect patient safety and the quality of nursing care [33], policies and measures that support AR symptom management are needed at the hospital level.

The present study found no significant association between AR and depressive symptoms. This finding contradicts that of a systematic review on the association between allergic disorders and the risk of depression that showed a higher risk of depression among patients with AR [34] and that of a Spanish cohort study that demonstrated an association between the severity of AR and depression [18]. In addition, Grosso et al. [20] reported that depression was significantly associated with a greater risk of AR symptom exacerbation. One possible reason for the inconsistency between the results of these previous studies and the present study may be differences in the severity of the AR symptoms. However, owing to the limitations in the data, the severity of the AR symptoms could not be identified in this study. Therefore, future studies on the severity of AR are warranted.

Finally, among the general characteristics of the participants, shift work was found to be significantly associated with sleep disturbances and fatigue. Previous studies have consistently reported that shift work negatively affects the extent of sleep disturbances and fatigue experienced among nurses [31,33,35]. The present study found that AR significantly affects sleep and fatigue, and shift work could be a factor contributing to sleep disturbance and fatigue among the nurses diagnosed with AR. Therefore, nurse managers should make more of an effort to reduce the harmful effects of shift work among nurses. In addition, measures should be taken to effectively reduce sleep disturbance and fatigue in nurses diagnosed with AR who perform shift work.

The present study found that AR negatively affects sleep and fatigue among Korean female nurses. Nurses can be exposed to various allergens, such as drugs, antiseptics, and latex gloves, at work, and such occupational exposures may aggravate their AR symptoms. Therefore, organizational support should be provided so that nurses are not unnecessarily exposed to allergens during work. Efforts made by nurse managers, such as to identify the detailed occupational history of nurses with AR and agents that nurses may be exposed to during work and to develop and implement safety guidelines for reducing harmful exposure [4,10], are considered forms of organizational support.

This study is noteworthy in that it is the first to examine the associations between AR and sleep disturbances, fatigue, and depression among female nurses. In addition, the results will aid in the development and implementation of health promotion activities among Korean nurses, because the study population included nurses working at hospitals nationwide. It may also be possible to extend these health promotion activities to females in the general population, because AR was found to be associated with sleep disturbances and fatigue even after controlling for the nature of nursing work. Despite its strengths, the present study had two limitations that should be addressed. First, the duration of the disease could not be determined due to limitations in the data used. Second, the present study did not identify the severity of the AR symptoms. Considering these limitations, future studies that investigate the effects of AR on sleep disturbances, fatigue, and depression are warranted.

## 5. Conclusions

The present study analyzed the associations between AR and sleep disturbances, fatigue, and depressive symptoms among Korean nurses. The results indicated that, after adjustment for the general and work-related characteristics of the participants, AR was significantly associated with sleep disturbances and fatigue but not with depressive symptoms. Nurses have high levels of sleep disturbance and fatigue due to the nature of their work compared to those in other occupations, and these issues are likely to be compounded in nurses with AR. Therefore, nurse managers should pay special attention to these situations and develop measures that prevent nurses diagnosed with AR from being unnecessarily exposed to allergens at work.

## Figures and Tables

**Table 1:** Baseline characteristics according to allergic rhinitis (*N* = 8645).

Characteristics	Category	No AR (*N* = 6837)		AR (*N* = 1808)		t or *x^2^*	*p*
		n	%	n	%		
Age, years	≤ 29	1217	17.8	327	18.1	10.16	0.017
	30–39	3829	56.0	1069	59.1		
	40–49	1651	24.1	373	20.6		
	≥ 50	140	2.0	39	2.2		
	M ± SD	35.58 ± 6.16		35.30 ± 5.85		1.82	0.069
Education level	3-year college	1235	18.1	339	18.8	2.55	0.280
	4-year college	4289	62.7	1151	63.7		
	Master or higher	1313	19.2	318	17.6		
Marital status BMI	Single or others	2551	37.3	646	35.7	1.54	0.218
	Married	4286	62.7	1162	64.3		
	Normal (18.5–23kg/m^2^)	4068	59.5	1037	57.4	5.35	0.069
	Underweight (<18.5kg/m^2^)	526	7.7	127	7.0		
	Overweight (≥23kg/m^2^)	2243	32.8	644	35.6		
	M ± SD	22.21 ± 3.24		22.41 ± 3.36		−2.23	0.026
Alcohol consumption	No	1928	28.2	487	26.9	1.13	0.302
	Yes	4909	71.8	1321	73.1		
Shift work	No	3512	51.4	953	52.7	1.03	0.315
	Yes	3325	48.6	855	47.3		
Atopic dermatitis	No	6478	94.7	1534	84.8	206.69	<0.001
	Yes	359	5.3	274	15.2		
Asthma	No	6777	99.1	1682	93.0	252.02	<0.001
	Yes	60	0.9	126	7.0		
Self-rated health	Good	2674	39.1	577	31.9	44.70	<0.001
	Fair	3316	48.5	923	51.1		
	Poor	847	12.4	308	17.0		

AR = Allergic Rhinitis, BMI = Body Mass Index, M = Mean, and SD = Standard Deviation.

**Table 2:** Sleep disturbance, fatigue, and depressive symptoms according to allergic rhinitis (*N* = 8645).

Characteristics	No AR (*N* = 6837)	AR (*N* = 1808)	t	*p*
	M ± SD	M ± SD		
Sleep disturbance	4.51 ± 4.34	5.42 ± 4.81	−7.28	<0.001
Fatigue				
Physical fatigue	11.01 ± 4.02	11.87 ± 3.74	−8.63	<0.001
Mental fatigue	5.15 ± 2.62	5.57 ± 2.63	−6.05	<0.001
Total	16.16 ± 6.02	17.44 ± 5.74	−8.39	<0.001
Depressive symptoms	4.81 ± 4.17	5.41 ± 4.33	−5.48	<0.001

AR = Allergic Rhinitis, M = Mean, and SD = Standard Deviation.

**Table 3:** Effects of allergic rhinitis on sleep disturbance (*N* = 8645).

Variables	Categories	Model 1			Model 2			Model 3			Model 4		
		β	t	*p*	β	t	*p*	β	t	*p*	β	t	*p*
Allergic rhinitis		0.08	7.74	<0.001	0.08	7.79	<0.001	0.07	6.38	<0.001	0.03	3.81	<0.001
Age, years					−0.08	−6.79	<0.001	−0.08	−6.65	<0.001	−0.03	−3.33	0.001
Education level	Master of higher				Ref.			Ref.			Ref.		
	3-year college				−0.01	−0.87	0.386	−0.01	−0.77	0.440	−0.01	−0.66	0.508
	4-year college				−0.02	−1.17	0.241	−0.02	−1.17	0.244	−0.01	−0.60	0.549
Marital status	Married				Ref.			Ref.			Ref.		
	Single or others				0.04	3.62	<0.001	0.04	3.54	<0.001	−0.01	−1.57	0.117
BMI					0.04	3.64	<0.001	0.04	3.43	0.001	0.01	1.53	0.125
Alcohol consumption	No				Ref.			Ref.			Ref.		
	Yes				0.03	2.81	0.005	0.03	2.87	0.004	0.02	2.40	0.016
Shift work	No				Ref.			Ref.			Ref.		
	Yes				0.12	11.34	<0.001	0.12	11.35	<0.001	0.07	7.99	<0.001
Atopic dermatitis	No							Ref.			Ref.		
	Yes							0.06	5.32	<0.001	0.03	3.73	<0.001
Asthma	No							Ref.			Ref.		
	Yes							0.03	2.46	0.014	0.01	1.49	0.135
Self-rated health	Good										Ref.		
	Fair										0.04	4.25	<0.001
	Poor										0.07	6.96	<0.001
Fatigue											0.09	7.95	<0.001
Depressive symptoms											0.48	41.65	<0.001
F(*p*)		59.84 (<0.001)			45.16 (<0.001)			39.74 (<0.001)			339.81 (<0.001)		
Adjusted R^2^		0.007			0.039			0.043			0.354		

BMI = Body Mass Index and Ref. = Reference.

**Table 4:** Effects of allergic rhinitis on fatigue (*N* = 8645).

Variables	Categories	Model 1			Model 2			Model 3			Model 4		
		β	t	*p*	β	t	*p*	β	t	*p*	β	t	*p*
Allergic rhinitis		0.09	8.16	<0.001	0.09	8.25	<0.001	0.08	7.30	<0.001	0.03	4.27	<0.001
Age, years					−0.04	−3.48	0.001	−0.04	−3.38	0.001	0.00	0.37	0.712
Education level	Master of higher				Ref.			Ref.			Ref.		
	3-year college				0.00	−0.34	0.737	0.00	−0.28	0.782	0.00	−0.34	0.732
	4-year college				−0.02	−1.69	0.090	−0.02	−1.69	0.091	−0.02	−1.63	0.103
Marital status	Married				Ref.			Ref.			Ref.		
	Single or others				−0.03	−3.02	0.003	−0.04	−3.08	0.002	−0.11	−13.18	<0.001
BMI					−0.03	−2.74	0.006	−0.03	−2.87	0.004	−0.07	−9.14	<0.001
Alcohol consumption	No				Ref.			Ref.			Ref.		
	Yes				0.01	0.85	0.394	0.01	0.88	0.378	0.00	0.31	0.756
Shift work	No				Ref.			Ref.			Ref.		
	Yes				0.09	8.36	<0.001	0.09	8.36	<0.001	0.02	2.93	0.003
Atopic dermatitis	No							Ref.			Ref.		
	Yes							0.04	3.31	0.001	0.00	0.59	0.554
Asthma	No							Ref.			Ref.		
	Yes							0.02	1.43	0.153	0.00	−0.43	0.664
Self-rated health	Good										Ref.		
	Fair										0.20	23.23	<0.001
	Poor										0.22	23.72	<0.001
Sleep disturbance											0.08	7.95	<0.001
Depressive symptoms											0.52	51.86	<0.001
F(*p*)		66.58 (<0.001)			21.44 (<0.001)			18.49 (<0.001)			533.39 (<0.001)		
Adjusted R^2^		0.008			0.019			0.020			0.463		

BMI = Body Mass Index and Ref. = Reference.

**Table 5:** Effects of allergic rhinitis on depressive symptoms (*N* = 8645).

Variables	Categories	Model 1			Model 2			Model 3			Model 4		
		β	t	*p*	β	t	*p*	β	t	*p*	β	t	*p*
Allergic rhinitis		0.06	5.48	<0.001	0.06	5.54	<0.001	0.05	4.53	<0.001	−0.01	−1.87	0.062
Age, years					−0.10	−7.95	<0.001	−0.10	−7.85	<0.001	−0.05	−5.54	<0.001
Education level	Master of higher				Ref.			Ref.			Ref.		
	3-year college				−0.01	−0.60	0.548	−0.01	−0.53	0.593	0.00	−0.14	0.888
	4-year college				−0.02	−1.18	0.238	−0.02	−1.17	0.240	0.00	0.02	0.982
Marital status	Married				Ref.			Ref.			Ref.		
	Single or others				0.11	9.86	<0.001	0.11	9.80	<0.001	0.11	13.60	<0.001
BMI					0.04	3.95	<0.001	0.04	3.80	<0.001	0.04	4.74	<0.001
Alcohol consumption	No				Ref.			Ref.			Ref.		
	Yes				0.02	2.05	0.040	0.02	2.09	0.037	0.01	1.12	0.264
Shift work	No				Ref.			Ref.			Ref.		
	Yes				0.08	7.50	<0.001	0.08	7.50	<0.001	−0.01	−0.74	0.462
Atopic dermatitis	No							Ref.			Ref.		
	Yes							0.04	3.65	<0.001	0.00	0.12	0.907
Asthma	No							Ref.			Ref.		
	Yes							0.02	1.85	0.065	0.00	0.17	0.865
Self-rated health	Good										Ref.		
	Fair										0.00	−0.51	0.611
	Poor										0.06	6.74	<0.001
Sleep disturbance											0.35	41.65	<0.001
Fatigue											0.46	51.86	<0.001
F(*p*)		29.98 (<0.001)			51.94 (<0.001)			43.32 (<0.001)			690.17 (<0.001)		
Adjusted R^2^		0.003			0.045			0.047			0.527		

BMI = Body Mass Index and Ref. = Reference.

## Data Availability

The datasets generated and analyzed during the current study are not publicly available, because this government data needs time for data clearing and the establishment of guidelines. The Korea Centers for Disease Control and Prevention is planning on opening this data to the public in the future.
